# Potential Surgical Implications of Internal Jugular Stenosis in a Craniocervical Junction Meningioma

**DOI:** 10.7759/cureus.26403

**Published:** 2022-06-28

**Authors:** Catherine Zhang, Lauren Harris, Hamza Itum, Sanjiv Chawda, Julian Coker, Jonathan Pollock, Ahmed-Ramadan Sadek, Alireza Shoakazemi

**Affiliations:** 1 Neurosurgery, Queen’s Hospital, Romford, GBR; 2 Neuroradiology, Queen’s Hospital, Romford, GBR; 3 Vascular Surgery, Queen’s Hospital, Romford, GBR

**Keywords:** posterior cervical surgery, eagle syndrome, craniocervical junction, meningioma, internal jugular vein stenosis

## Abstract

We report a case of a 61-year-old lady presenting with several weeks of progressive left-sided weakness, and found to have a foramen magnum meningioma. She was counselled on surgical resection of the tumour, and a preoperative computed tomography angiogram (CTA) was obtained for operative planning purposes. CTA demonstrated incidental bilateral internal jugular vein (IJV) stenosis, with enlarged extracranial collateral vessels and elongated styloid processes. The main surgical concern was potential injury of the extracranial collateral vessels during operative exposure, which may compromise her intracranial venous outflow in light of the IJV stenosis. A doppler ultrasound scan of the IJVs was performed, which demonstrated that blood flow was still present through both vessels. Through careful soft tissue dissection during surgery, potential complications and injury to the extracranial collaterals were avoided.

We performed a literature review of the incidence of IJV stenosis, its associated conditions, and potential surgical implications. Complications from injury to vital collateral extracranial vessels should be considered during preoperative planning in patients with anatomical variants or risk factors for IJV stenosis, as seen in this case.

## Introduction

Internal jugular vein (IJV) stenosis is being increasingly associated with certain neurological conditions, including idiopathic intracranial hypertension (IIH), cerebrospinal fluid (CSF) disorders, multiple sclerosis, and Alzheimer’s disease [1]. The role of surgery in IJV stenosis has typically been to manage the associated CSF disorders and/or the insertion of IJV stents. We describe a case of a patient incidentally found to have bilateral IJV stenosis with engorged paraspinal venous collaterals during preoperative computed tomography angiogram (CTA) for a symptomatic craniocervical junction meningioma. We discuss the surgical implications of these findings and review the literature on aetiology and clinical phenomena associated with IJV stenosis.

## Case presentation

We report a case of a 61-year-old right-handed lady who presented to a local accident and emergency department with a several-week history of gradual heaviness affecting her left arm, on a background of 4 months of left-sided paraesthesia. She had no right-sided symptoms.

On further questioning, she also described frequent coughing, difficulty clearing her throat and reaching high-pitched sounds during singing, and a few weeks of dysphagia. Her past medical history included acute idiopathic thrombocytopenic purpura, complicated by pneumonia 10 years previously. She is a keen horse-rider. On examination, she had a normal gait, with negative Romberg and Unterberger’s tests. There was a reduced sensation of light touch in her left hand. Her tongue was symmetrical, with mild fasciculations in the neutral position, and she had a slight uvular deviation to the right. Examination of lower cranial never function by flexible naso-endoscopy did not reveal any abnormality.

**Figure 1:**
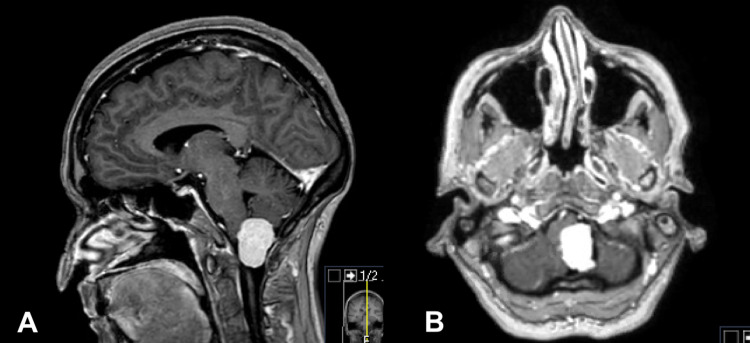
Post-contrast sagittal (A) and axial (B) MRI scans. The scans demonstrating a 23×16×22 mm intradural, extramedullary lesion with medullary and upper cervical cord compression.

CT scan demonstrated a left-sided mass at the level of the foramen magnum, measuring 3×16×22 mm, with brainstem compression. Magnetic resonance imaging (MRI) demonstrates a homogenously contrast-enhancing intradural, extramedullary lesion, causing compression of the medulla and upper cervical spinal cord (Figure 1).

**Figure 2:**
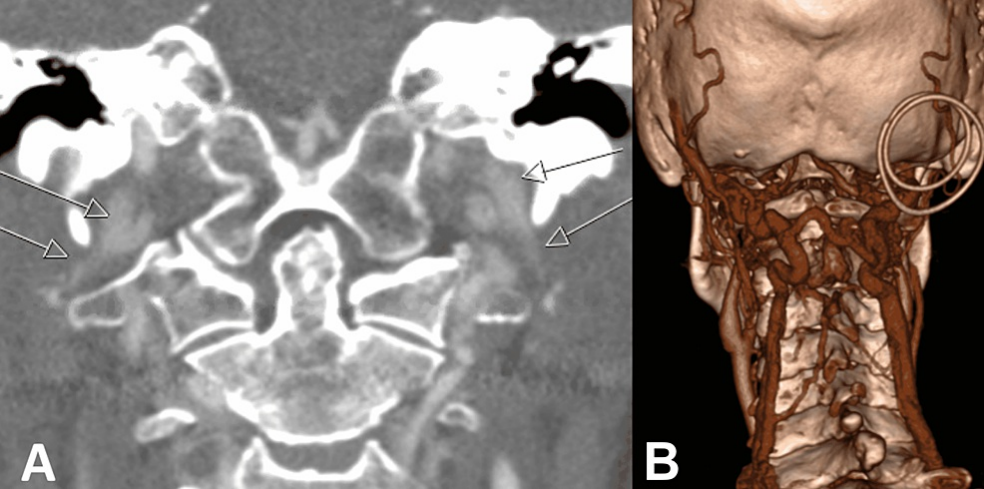
Coronal view of CT angiogram. The scans demonstrating bilateral narrowing of IJVs at the junction between the styloid processes and C1 lateral mass (arrows) (A). Large, tortuous, engorged anastomotic venous plexus crossing the midline, with collaterals to the posterior jugular vein and bilateral vertebral veins (B). IJV: internal jugular vein

Initial differential diagnoses included meningioma or nerve sheath tumour. A brain and cervical spine CTA was performed as a part of routine preoperative planning. A review of this scan in the skull base multidisciplinary team (MDT) raised a possibility of bilateral jugular vein obstruction just below the skull base (Figure 2A), with enlarged deep venous collaterals in the cervical paraspinal muscles (Figure 2B).

**Figure 3:**
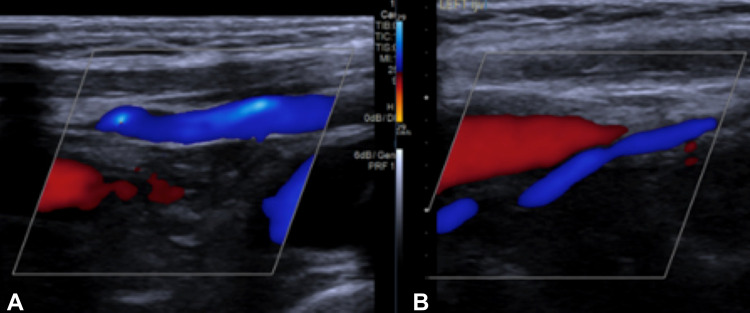
Right (A) and left (B) internal jugular vein doppler ultrasound showing normal venous flow.

There was also a finding of bilateral elongated styloid processes of 4 cm, with associated bilateral IJV stenosis. The reduced calibre of both IJVs continued throughout the neck, and the enlarged collateral vessels were seen to drain directly from the sigmoid sinuses. The concern at this stage was that the paraspinal venous channels were the primary intracranial venous outflow, with potential risks of venous hypertension, infarction and brain swelling in the event of venous injury during the surgical exposure. Following discussion with vascular surgery, a doppler ultrasound of the neck vessels was performed, which demonstrated patency and a reasonable blood flow through both IJVs. There was no evidence of underlying thrombosis (Figure 3).

Although this was reassuring, the patient was counselled in detail about small risks of potential venous infarction, surgical access difficulties and incomplete resection.

Surgical approach

**Figure 4:**
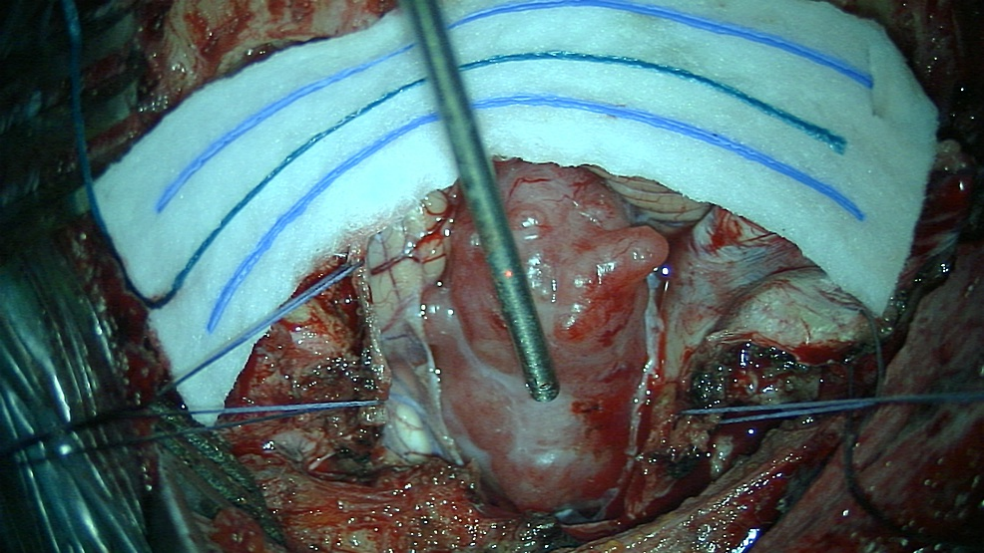
Intraoperative findings of a firm extra-axial tumour with a clear arachnoid plane indenting the cerebellar tonsils, hemisphere and brainstem.

A midline hockey-stick incision curving to the left was used, with careful dissection in the midline raphe. There was no breach of the deep cervical dilated veins on the surgical exposure. A left suboccipital craniectomy was performed, with C1 hemilaminectomy, and a part of the left occipital condyle was drilled down to facilitate surgical access. After applying the retractors, flow through deep veins was confirmed with intra-op ultrasonography. A hockey-stick dural incision was made. The tumour was firm, extra-axial, with a good arachnoid plane and was debulked internally to minimise retraction on adjacent structure (Figure 4). The dural entry point of the left vertebral artery was identified and seen to be separate from the dural base of the tumour. The tumour was then completely removed, and the dural base coagulated, giving a Simpson Grade 2 resection. Dura was closed with dural substitute reinforcement and sealant glue. The cervical fascia was closed in layers, with no significant venous bleeding. Intraoperative neuromonitoring was used throughout the procedure, which showed no changes in motor-evoked potentials (MEPs), somatosensory-evoked potentials (SSEPs), corticobulbar motor-evoked potentials (coMEPs) of lower cranial nerves, brainstem auditory-evoked potentials (BAEPs) and laryngeal adductor reflex (LAR). Spinal accessory and hypoglossal nerves were visualised and protected throughout the surgery.

**Figure 5:**
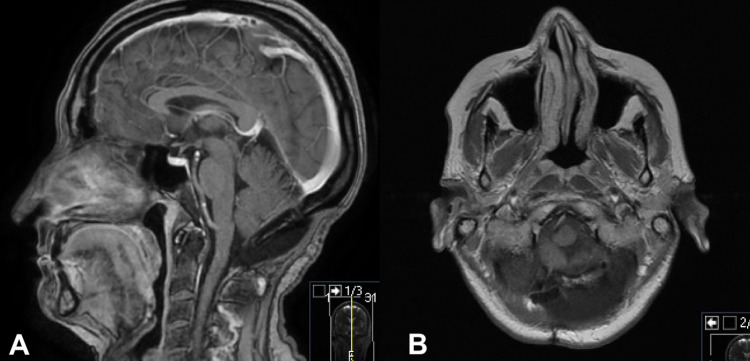
Postoperative MRI scan. The scans in sagittal (A) and axial (B) planes showing gross-total resection.

The patient was discharged home on postoperative day 5 with no neurological deficits. Immediate postoperative MRI showed no residual tumour (Figure 5), and histology was that of a WHO Grade 1 meningothelial meningioma. Postoperative vascular imaging demonstrated no change in the venous structures in paraspinal musculature. At six-month follow-up, the patient’s left upper limb symptoms had resolved and she made a complete neurological recovery. She developed a right femoral pseudoaneurysm secondary to preoperative venous catheterisation, and this was resolved with conservative management. She reported no further symptoms concerning IJV stenosis or venous outflow obstruction.

## Discussion

IJVs are the main intracerebral venous outflow channels and have valves to prevent jugular reflux and raised intracranial pressure. Collateral vessels exist between both of the IJVs to regulate venous outflow and also anastomoses with the internal and external vertebral venous plexus through condylar veins at the anterior condylar confluence [2]. Multiple studies have also previously described the vertebral venous plexus as an important collateral intracranial venous drainage [2,3].

Causes of internal jugular venous stenosis include intraluminal anomalies such as membranes and webs as well as focal thrombi. Extraluminal causes include anatomical variations causing direct compression of the IJV along its course, typically from elongated styloid processes, enlarged C1 transverse process, thyroid goitres, mediastinal masses or adjacent enlarged arteries. The most commonly stenosed region is at the upper jugular J3 segment [4]. Little is published in the literature on the true incidence of IJV stenosis; however, they have recently been implicated in a number of neurological conditions. They are well known to be associated with IIH and disrupt CSF dynamics [5,6]. A recent review article also suggests a possible association between IJV stenosis and conditions such as multiple sclerosis and Alzheimer’s disease [1,7,8]. Typical associated symptoms include headache, tinnitus, visual disturbances and also intracranial hypertension or cerebral haemorrhage.

One study attempted to quantify the radiological incidence of IJV stenosis in 108 patients who had CT neck angiograms as investigations for traumatic brain injury, atherosclerotic disease or neck dissections [9]. In this study of 108 patients, the incidence of IJV stenosis from direct external compression was up to 33%. 16.7% had severe dominant IJV stenosis (i.e., >80%), of which approximately 55% were associated with extracranial condylar collateral vessels. The most common causes of IJV stenosis were elongated styloid process and enlarged digastric muscles.

The patient described in our case report did have bilaterally elongated styloid processes of 4 cm and there was a significant narrowing of the IJV calibre bilaterally in between the styloid process and C1 lateral masses, which raised the question of Eagle Syndrome. Elongated styloid processes exist in approximately 4% of the population and can have a myriad of clinical manifestations. Symptomatic elongated styloid processes (i.e., Eagle Syndrome) typically manifest with pain and dysphagia, usually after a tonsillectomy and formation of localised scar around the styloid process. When the IJV is compressed between the elongated styloid and C1 transverse process, “Eagle Jugular Syndrome” can arise, which can also incorporate intracranial hypertension symptoms [10-13]. The primary treatment for Eagle Jugular Syndrome is styloidectomy. On further retrospective questioning the patient in our case report, it was concluded that she did not have any clinical symptoms of Eagle Syndrome and most importantly the doppler scan confirmed adequate blood flow through both IJVs.

Surgical relevance

The surgical relevance of IJV stenosis pertinent to this case report is the presence of enlarged venous plexus collaterals within the cervical paraspinal muscles that were at risk of compromise in a posterior cervical approach. This was only picked up incidentally during a preoperative CT neck angiogram. The true incidence of intracerebral venous outflow obstruction is still unknown and is most likely underdiagnosed due to the associated nonspecific clinical symptoms. Based on Jayaraman et al.’s paper, the incidence of incidental dilated paraspinal venous collaterals secondary to IJV stenosis can be up to 8% [9]. Whilst doppler ultrasound excluded any intraluminal thrombi as the cause of our patient’s IJV stenosis and confirmed the presence of blood flow, it did not quantify the percentage of intracerebral venous outflow through the IJV and, therefore, how dependent the patient was on the collateral venous drainage. The exact pathophysiology at present is still unclear, and there may be a complex underlying process in this patient’s IJV stenosis. Scerrati’s review looking at symptomatic stylogenic cervical IJV stenosis found that only 1/3 of patients had elongated styloid processes, and stenosis is most likely a combination of both extraluminal and intraluminal factors [1].

Given the relatively high incidence of IJV stenosis and collateral vessels, and the increasing number of pathologies associated with IJV stenosis, damaging collateral venous drainage in a posterior cervical approach and potentially precipitating other associated neurological conditions should be considered during operative planning in patients with anatomical variants or risk factors.

## Conclusions

We described the case of a patient with incidental bilateral IJV stenosis and associated dilated deep cervical venous plexus, during preoperative investigations for a craniocervical junction meningioma, and discussed potential surgical implications. Patients with anatomical variants or risk factors for IJV stenosis and dilated deep cervical veins should be counselled on potential associated complications when undergoing posterior cervical approach surgeries.

## References

[1] Scerrati A, Norri N, Mongardi L (2021). Styloidogenic-cervical spondylotic internal jugular venous compression, a vascular disease related to several clinical neurological manifestations: diagnosis and treatment—a comprehensive literature review. Ann Transl Med.

[2] Carpenter K, Decater T, Iwanaga J, Maulucci CM, Bui CJ, Dumont AS, Tubbs RS (2021). Revisiting the vertebral venous plexus-A comprehensive review of the literature. World Neurosurg.

[3] Doepp F, Lammert I, Hoffmann O, Einhäupl KM, Schreiber S, Valdueza JM (2001). Venous collateral blood flow assessed by Doppler ultrasound after unilateral radical neck dissection. Ann Otol Rhinol Laryngol.

[4] Zhou D, Ding J-Y, Ya J-Y (2018). Understanding jugular venous outflow disturbance. CNS Neurosci Ther.

[5] Nedelmann M, Kaps M, Mueller-Forell W (2009). Venous obstruction and jugular valve insufficiency in idiopathic intracranial hypertension. J Neurol.

[6] Ball AK, Clarke CE (2006). Idiopathic intracranial hypertension. Lancet Neurol.

[7] Zamboni P, Galeotti R, Salvi F (2020). Effects of venous angioplasty on cerebral lesions in multiple sclerosis: expanded analysis of the brave dreams double-blind, sham-controlled randomized trial. J Endovasc Ther.

[8] Laupacis A, Lillie E, Dueck A (2011). Association between chronic cerebrospinal venous insufficiency and multiple sclerosis: a meta-analysis. CMAJ.

[9] Jayaraman MV, Boxerman JL, Davis LM, Haas RA, Rogg JM (2012). Incidence of extrinsic compression of the internal jugular vein in unselected patients undergoing CT angiography. Am J Neuroradiol.

[10] Zamboni P, Scerrati A, Menegatti E (2019). The eagle jugular syndrome. BMC Neurol.

[11] Bai C, Wang Z, Guan J, Jin K, Ding Y, Ji X, Meng R (2020). Clinical characteristics and neuroimaging findings in eagle syndrome induced internal jugular vein stenosis. Ann Transl Med.

[12] Li M, Sun Y, Chan CC, Fan C, Ji X, Meng R (2019). Internal jugular vein stenosis associated with elongated styloid process: five case reports and literature review. BMC Neurol.

[13] Fusco DJ, Asteraki S, Spetzler RF (2012). Eagle's syndrome: embryology, anatomy, and clinical management. Acta Neurochir.

